# p53 mutations as a marker of malignancy in bladder washing samples from patients with bladder cancer

**DOI:** 10.1054/bjoc.1999.0890

**Published:** 1999-12-08

**Authors:** H A Phillips, G C W Howard, W R Miller

**Affiliations:** Department of Clinical Oncology, NHS; Department of Clinical Oncology, University of Edinburgh, Western General Hospital, Edinburgh, UK

**Keywords:** p53, mutations, bladder cancer, bladder washings

## Abstract

The diagnosis and follow-up of patients with bladder cancer rely on invasive procedures (cystoscopy with biopsy). Polymerase chain reaction (PCR)-based technologies may allow the sensitive detection of cancer-related genetic mutations in exfoliated tumour material, potentially allowing the development of less invasive techniques. This pilot study investigated the feasibility of detecting mutations in exons 5–8 of the p53 gene using single-stranded conformational polymorphism (SSCP) analysis in bladder-washing specimens from patients with bladder cancer. Bladder-washing samples (31) were collected from patients (27) with bladder cancer. An abnormal additional SSCP band was detected in five samples from five different patients suggesting the presence of a p53 mutation. In all five cases the same abnormal SSCP pattern was demonstrated in samples of the corresponding bladder tumour. In one case bladder washings were available from the same patient on two separate occasions with one washing demonstrating a mutation and the other not. In two further cases a mutation was demonstrated in the bladder tumour but not in the corresponding washing. It is concluded that it is possible to detect and characterize p53 mutations in bladder-washing samples from patients with bladder cancer. Improved sensitivity in detecting mutations in a sample containing a mixture of normal and malignant cells may lead to the clinical applicability of molecular methods of disease monitoring. © 2000 Cancer Research Campaign


*British Journal of Cancer* (2000) **82**(1), 136–141
© 2000 Cancer Research Campaign
Article no. bjoc.1999.0890

### p53 mutations as a marker of malignancy in bladder washing samples from patients with bladder cancer

**HA Phillips[1,2], GCW Howard[1,2] and WR Miller[2]**

[1]NHS Department of Clinical Oncology, [2]University of Edinburgh, Department of Clinical Oncology, Western General Hospital, Edinburgh, UK

**Summary** The diagnosis and follow-up of patients with bladder cancer rely on invasive procedures (cystoscopy with biopsy). Polymerase chain reaction (PCR)-based technologies may allow the sensitive detection of cancer-related genetic mutations in exfoliated tumour material, potentially allowing the development of less invasive techniques. This pilot study investigated the feasibility of detecting mutations in exons 5–8 of the p53 gene using single-stranded conformational polymorphism (SSCP) analysis in bladder-washing specimens from patients with bladder cancer. Bladder-washing samples (31) were collected from patients (27) with bladder cancer. An abnormal additional SSCP band was detected in five samples from five different patients suggesting the presence of a p53 mutation. In all five cases the same abnormal SSCP pattern was demonstrated in samples of the corresponding bladder tumour. In one case bladder washings were available from the same patient on two separate occasions with one washing demonstrating a mutation and the other not. In two further cases a mutation was demonstrated in the bladder tumour but not in the corresponding washing. It is concluded that it is possible to detect and characterize p53 mutations in bladder-washing samples from patients with bladder cancer. Improved sensitivity in detecting mutations in a sample containing a mixture of normal and malignant cells may lead to the clinical applicability of molecular methods of disease monitoring. © 2000 Cancer Research Campaign

**Keywords**: p53; mutations; bladder cancer; bladder washings

The diagnosis and follow-up of patients with bladder cancer relies heavily on the use of invasive procedures, most commonly cystoscopy with biopsy. Malignant cells are shed into the bladder but the use of urine cytology has well documented limitations in the diagnosis and monitoring of bladder cancer most particularly in relation to sensitivity (Maier et al, 1995). A number of strategies have been adopted to enhance the diagnostic potential of urine samples to lessen the reliance on invasive procedures. Some investigators have studied bladder-washing samples which have the advantage over voided urine samples of increased cellularity (Walther, 1992). Recently interest has developed in the use of cancer-associated genetic abnormalities as markers for the presence of malignancy in such samples (Sidransky et al, 1991; Levesque et al, 1993; Fitzgerald et al, 1995).

p53 mutations are the most common acquired or somatic genetic abnormalities in human malignant disease (Hollstein et al, 1991) and occur frequently in bladder cancer. Mutations are most common in high-grade and invasive bladder tumours (Fujimoto et al, 1992) with up to 40–60% of muscle-invasive tumours carrying mutations (Sidransky et al, 1991; Fujimoto et al, 1992; Esrig et al, 1993). Most p53 mutations are missense point mutations and approximately 80% are found in the evolutionarily highly conserved and functionally important exons 5–8 of the gene (Nigro et al, 1989; Hollstein et al, 1991).

*Received 24 February 1999*
*Revised 22 June 1999*
*Accepted 7 July 1999*

*Correspondence to:* HA Phillips, Dept of Clinical Oncology, Western General Hospital, Crewe Road, Edinburgh EH4 2XU, UK

Sidransky et al (1991) were the first to describe p53 mutations in bladder cancer – mutations were identified in nine out of a cohort of 16 bladder cancers. Additionally, mutation-specific oligonucleotides were used to demonstrate the presence of mutations in corresponding urine samples in three cases, and it was estimated that 1–7% of cells in these urine samples harboured the relevant mutation.

Because of their relative frequency in bladder cancer, p53 mutations were chosen to explore further the diagnostic potential of molecular markers in this disease. The current study investigated whether p53 mutations could be identified and characterized in bladder washing samples from patients with bladder cancer without prior knowledge of the mutation being sought. Exons 5–8 of the p53 gene were screened for the presence of mutations using single-strand conformational polymorphism (SSCP) analysis and when available corresponding bladder tumour specimens were subsequently studied in the same way. Samples with abnormal SSCP patterns were subjected to direct sequencing.

The study was undertaken with the approval of the Lothian Research Ethics Committee.

### MATERIALS AND METHODS

### Subjects

Thirty-one bladder-washing samples were collected from 27 patients with bladder cancer, bladder washings being collected from four patients on two separate occasions. In each case a bladder abnormality was seen at cystoscopy. On 29 occasions a bladder tumour was present. In two patients with previously documented bladder cancer the biopsy taken at the time of bladder washing was

**Table 1** Clinicopathological characteristics of tumours

| | |
|---|---|
| Histology | |
| Transitional cell carcinoma | 28 |
| Squamous cell carcinoma | 1 |
| Mixed squamous TCC | 2 |
| Grade | |
| Grade 1 (well differentiated) | 4 |
| Grade 2 (moderately differentiated) | 4 |
| Grade 3 (poorly differentiated) | 21 |
| Not applicable | 2 |
| Stage | |
| pTa (superficial papillary) | 8 |
| pT1 (invades lamina propria) | 5 |
| pT2 (invades muscle) | 14 |
| pTx | 2 |
| CIS previous pT1 | 1 |
| Dysplasia previous pTa | 1 |

abnormal, showing in one case carcinoma in situ and in the other case dysplastic urothelium. Fresh bladder tumour biopsy specimens were collected on 23 occasions from 20 of the above patients at the time of bladder washing sample collection. The clinicopathological characteristics of the subjects are shown in Table 1.

### Sample collection

Bladder-washing samples were collected at the time of cystoscopy, prior to any tumour resection (Hermansen et al, 1988). Normal saline (50–60 ml) was repeatedly (3–5 times) injected into and aspirated from the bladder. The resultant retrieved fluid was split between two sterile containers and centrifuged at 2000 rpm for 10 min. The cellular pellets were then stored in liquid nitrogen. When available a sample of resected bladder tumour was immediately snap-frozen in liquid nitrogen.

### DNA preparation

DNA was extracted from bladder-washing samples and both fresh and paraffin-embedded bladder tumour specimens using the QIA amp tissue kit (Qiagen UK Ltd) following the manufacturer's instructions.

### PCR-SSCP

A $^{32}$P radiolabelled PCR-SSCP assay was established based on the method of Orita et al (1989). Four pairs of intronic primers were designed to amplify the whole of each of exons 5–8 of the p53 gene. Primer sequences, product size and primer annealing temperatures are shown in Table 2.

### PCR

PCR reactions were performed in a 20 μl volume containing 1 μl DNA (approx 100 ng) as template, 2 μl 10× reaction buffer (Promega, UK), 2 μl 2 mM dNTPs, magnesium chloride 1 mM, 0.2 μl of each of a primer pair (200 ng $\mu l^{-1}$), 1.5 units PIC/*Taq* DNA polymerase (ICRF Central Services, London, UK), 0.074 MBq $\alpha^{32}$P dCTP (Amersham, UK), made up with distilled water. All reactions contained a no template control, made up last, to ensure the integrity of the reaction. PCR cyling was as follows: 94°C for 7 min, 35 cycles of 94°C for 1 min, primer annealing temperature for 1 min (Table 2) and 72°C for 1 min; with a final 10 min at 72°C.

### SSCP

Aliquots (2 μl) from the PCR reaction were mixed with 10 μl of SSCP dye (95% formamide, 5 mM sodium hydroxide, 0.1% bromophenol blue, 0.1% xylene cyanol), heated to 95°C for 5 min, immediately chilled on ice and approximately 6 μl loaded per well of a non-denaturing polyacrylamide gel. To increase the sensitivity of the screen, all samples were run under three sets of gel conditions (firstly 8% acrylamide, 2% bisacrylamide with 10% glycerol run at 4°C and secondly 10% acrylamide, 1.3% bisacrylamide with 10% glycerol run at room temperature for all four exons studied; finally either 8% acrylamide, 2% bisacrylamide with no glycerol run at 4°C [exons 7 and 8] or 10% acrylamide, 1.3% bisacrylamide with no glycerol run at 4°C [exons 5 and 6]). Gels were dried and subjected to autoradiography. Reactions were only accepted if the no template control yielded no product.

### Direct DNA sequencing

DNA sequencing was performed using Sequenase Version 2.0 DNA sequencing kit (Amersham) as previously described (Winship, 1989; Prosser et al, 1990). The sequencing template was obtained from a non-radiolabelled PCR reaction of the relevant exon of the sample of interest using either sample DNA or (if the mutation was present as a small proportion of the sample) abnormal single-stranded DNA eluted from an abnormal band excised from a dried SSCP gel.

### RESULTS

### p53 mutations in bladder-washing specimens

Thirty-one bladder-washing samples from 27 patients were studied. Exons 5–8 of the p53 gene were screened for the presence of mutations. Five samples each from separate patients showed an

**Table 2** PCR primers

| | Primer sequence | Region amplified | Product size | Annealing temperature |
|---|---|---|---|---|
| Exon 5 Sense | 5′TGTGCCCTGACTTTCAACTC | 42 bases upstream to 37 bases | 263 base pairs | 57°C |
| Antisense | 5′AACCAGCCCTGTCGTCTCTC | Downstream of exon 5 | | |
| Exon 6 Sense | 5′TGATTCCTCACTGATTGCTC | 24 bases upstream to 20 bases | 157 base pairs | 53°C |
| Antisense | 5′ACCCCAGTTGCAAACCAGAC | Downstream to exon 6 | | |
| Exon 7 Sense | 5′AAGGCGCACTGGCCTCATCT | 40 bases upstream to 30 bases | 181 base pairs | 59°C |
| Antisense | 5′CAGTGTGCAGGGTGGCAAGT | Downstream of exon 7 | | |
| Exon 8 Sense | 5′GGACCTGATTTCCTTACTGC | 50 bases upstream to 54 bases | 241 base pairs | 57°C |
| Antisense | 5′GAGGCATAACTGCACCCTTG | Downstream of exon 8 | | |

© 2000 Cancer Research Campaign 

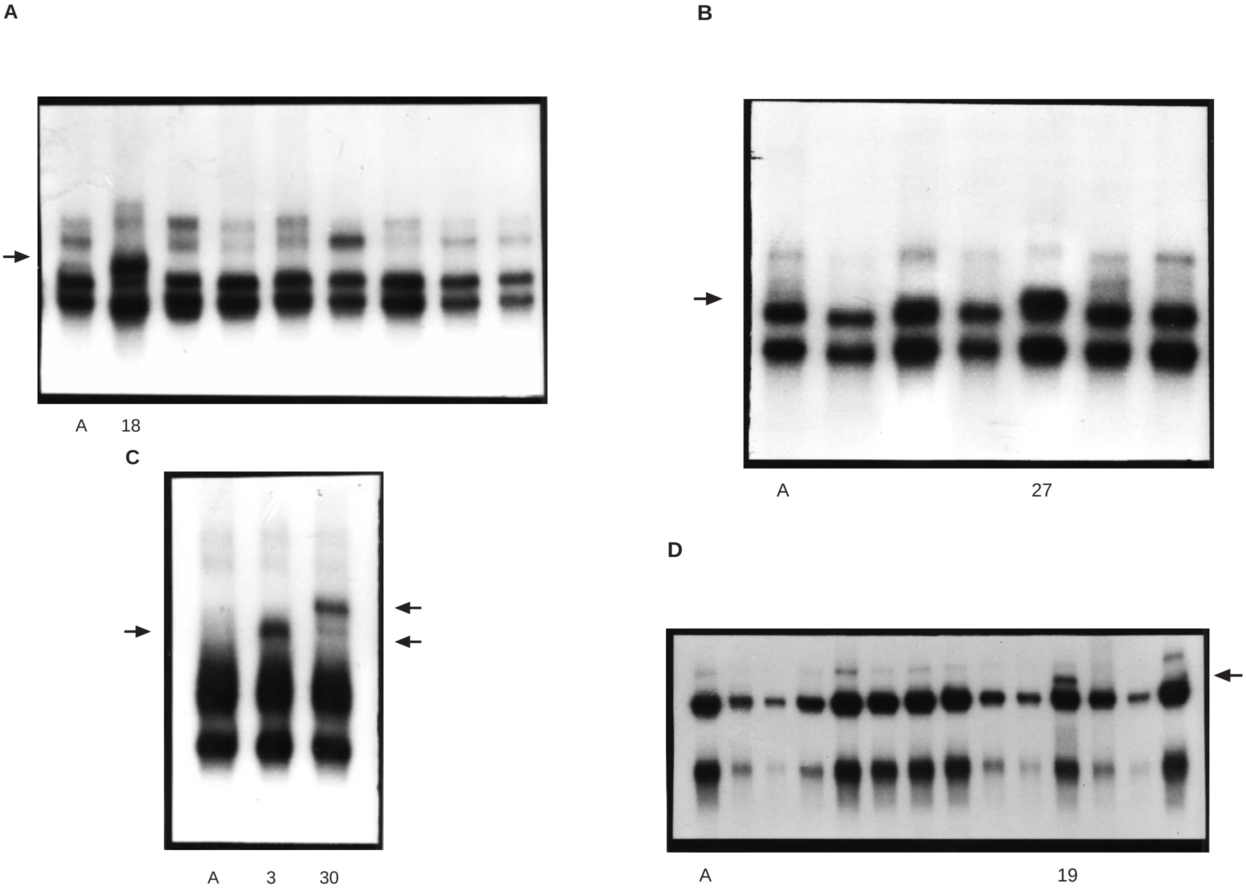

**Figure 1** Autoradiographs of SSCP gels demonstrating abnormal migration patterns consistent with the presence of mutations in five out of 31 bladder washing samples from patients with bladder cancer (abnormalities are indicated by arrows).(**A**) Exon 6: an abnormal extra band is seen in sample 18; (**B**) Exon 6: an abnormal extra band is seen in sample 27; (**C**) Exon 7: one abnormal extra band is seen in sample 3 and two extra bands in sample 30; (**D**) Exon 8: an abnormal extra band is seen in sample 19. Lanes marked A show A2780 cell line normal controls (Brown et al, 1993)

abnormal SSCP pattern consistent with the presence of a mutation (Figure 1). Of these mutations two were in exon 6, two in exon 7 and one in exon 8. Of the abnormalities found two were identifiable in all three sets of gel conditions used, two were seen in two sets of conditions and one in only one, emphasizing the importance of using more than one set of gel to enhance sensitivity (data not shown).

### Correlation between mutation detection in surgical biopsy/resection samples and bladder-washing samples

The concordance between the SSCP results obtained in bladder-washing samples and those obtained from analysis in the same manner of tumour tissue was evaluated. Twenty-three pairs of bladder-washing and tumour samples (obtained at the same cystoscopy) were available from a total of 20 patients, three patients having pairs of samples taken on two different occasions. The results are shown in Table 3. Concordant results (i.e. the presence or absence of an SSCP abnormality in both washing and tumour) were found on 19/23 occasions (83%). In one patient two pairs of samples showed an abnormality in the tumour samples but in neither of the washings. In one case two pairs of samples were again available and whilst both tumour specimens had an abnormal SSCP only one of the two washings demonstrated it. In a final patient an abnormality detected in tumour was again not detected in the corresponding bladder-washing specimen (Figure 2). It is important to note that these results include three samples in which the fresh bladder tumour specimen corresponding to SSCP abnormal bladder-washing samples failed to demonstrate a mutation. This was felt likely to represent poor tumour sampling and therefore further DNA was extracted, this time from paraffin-embedded tissue containing histologically confirmed tumour. When these repeat samples were analysed they each demonstrated the same SSCP abnormalities as the corresponding bladder-washing samples. It remains possible therefore that in further bladder tumour samples mutations were missed due to poor sampling – paraffin-embedded tissue only being studied in the three patients described above.

### Confirmation of mutations detected by SSCP using direct sequencing

The mutation present in 6 of the abnormal samples from four patients were confirmed by direct sequencing (Table 4) and examples shown in Figure 3. It did not prove possible to characterize the putative mutation in three samples with exon 7 abnormalities due

 © 2000 Cancer Research Campaign

**Table 3** Correlation between SSCP abnormalities detected in bladder washing and tumour samples

| Patient | Sample | Tumour pathology | | Tumour SSCP | Bladder wash SSCP | Correlate |
|---|---|---|---|---|---|---|
| A | 3 | G3pTC | TCC | *Abnormal Exon 7* | Abnormal Exon 7 | Yes |
| B | 6 | G3pTC | TCC | Normal | Abnormal Exon 7 | Yes |
| C | 7 | G2pTa | TCC | Normal | Normal | Yes |
| D | 8 | G2pTa | TCC | Normal | Normal | Yes |
| E1 | 9 | G3pT2 | TCC/Sq | Abnormal Exon 8 | Normal | No |
| E2 | 24 | G3pT1 | TCC/Sq | Abnormal Exon 8 | Normal | No |
| G1 | 12 | G3pT2 | TCC | Normal | Normal | Yes |
| G2 | 33 | G3pT2 | TCC | Normal | Normal | Yes |
| H | 13 | G3pT2 | TCC | Normal | Normal | Yes |
| I | 14 | G3pT2 | TCC | Normal | Normal | Yes |
| J | 15 | G3pT2 | TCC | Normal | Normal | Yes |
| K | 16 | G3pT2 | Sq | Normal | Normal | Yes |
| M1 | 18 | G3pT1 | TCC | Abnormal Exon 6 | Abnormal Exon 6 | Yes |
| M2 | 26 | G3pTa | TCC | Abnormal Exon 6 | Normal | No |
| N | 19 | G3pT2 | TCC | Abnormal Exon 8 | Abnormal Exon 8 | Yes |
| O | 20 | G2pT1 | TCC | Normal | Normal | Yes |
| Q | 22 | G3pTa | TCC | Normal | Normal | Yes |
| S | 25 | G2pTx | TCC | Normal | Normal | Yes |
| T | 27 | G3pT1 | TCC | *Abnormal Exon 6* | Abnormal Exon 6 | Yes |
| U | 28 | G1pTa | TCC | Normal | Normal | Yes |
| W | 30 | G3pT2 | TCC | *Abnormal Exon 7* | Abnormal Exon 7 | Yes |
| Y | 40 | G1pTa | TCC | Normal | Normal | Yes |
| Z | 41 | G3pT2 | TCC | Abnormal Exon 7 | Normal | No |

Patients from whom two pairs of samples were collected are indicated 1 and 2. Tumour samples in italics indicate those samples were an SSCP abnormality was detected in paraffin-embedded tumour but not the original fresh tumour studied. TCC: Transitional carcinoma; Sq: Squamous cell carcinoma; pT: pathological tumour stage; G: histological grade.

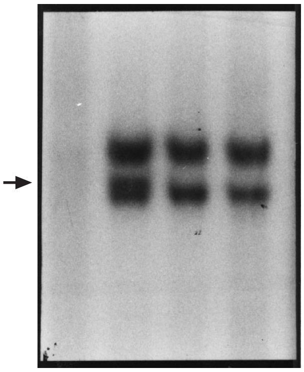

**Figure 2** Autoradiograph of SSCP gel illustrating the presence of an abnormal extra band (→) indicating the presence of a mutation in the bladder tumour sample from patient Z (41T) but not in the corresponding bladder washing sample (41W) nor in the A2780 cell line normal control (A)

**Table 4** Mutations confirmed by direct sequencing

| Patient | Sample(s) | Exon | Wild-type | Mutation | Codon |
|---|---|---|---|---|---|
| E | 9 and 24 | 8 | GAC(Asp) | AAC(Asn) | 281 |
| M | 18 and 26 | 6 | TAG | TAA | Upstream splice site |
| N | 19 | 8 | AGA(Arg) | ACA(Thr) | 280 |
| T | 27 | 6 | GAG(Glu) | GCG(Ala) | 224 |

to the technical failure to reamplify a double-stranded DNA template for sequencing from single-stranded DNA eluted from abnormal bands excised from dried SSCP gels. However, the consistently reproducible nature of the SSCP pattern detected for these samples strongly supports the presence of mutation rather than SSCP artifact.

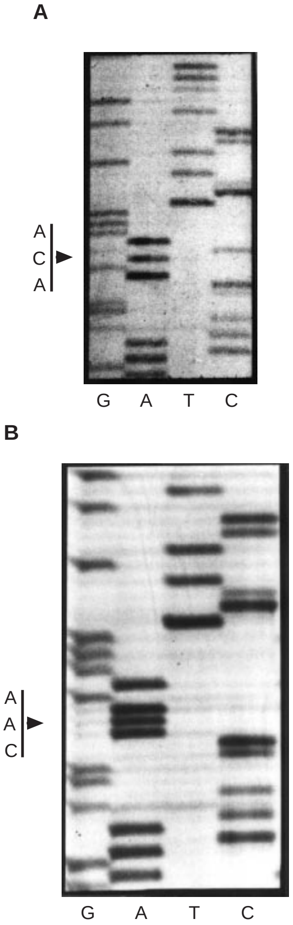

**Figure 3** Sequencing gels demonstrating: (**A**) a AGA-ACA mutation at codon 280 (exon 8) in samples from patient N; (**B**) a GAC-AAC mutation at codon 281 (exon 8) in samples from patient E

*© 2000 Cancer Research Campaign* 

### DISCUSSION

Using standard methodologies it has been possible to detect and characterize p53 mutations in a proportion of bladder-washing samples from patients with bladder cancer. This detection did not depend on prior knowledge of the mutation detected. Furthermore, the presence of the same abnormalities could be demonstrated in tumour biopsy or resection specimens.

However, there were four instances in which a mutation was demonstrated in the tumour specimen but not in the corresponding bladder-washing sample. Such false-negative bladder-washing results are a concern and limit the clinical applicability of such an assay using the methods employed in this study. As all patients studied in the current investigation had visible cystoscopic abnormalities it seems likely that at least some tumour cells were shed into the bladder-washing sample. The occurrence of false-negative bladder-washing results is therefore most readily attributable to the limited sensitivity of SSCP in detecting a mutation when the mutation-carrying cells account for only a small proportion of the sample. Sidransky et al (1991) estimated that only 1–7% of cells in the urine samples of three patients with bladder cancer carried a mutation. Such a level overlaps with the limits of sensitivity we have defined for SSCP (Phillips et al, 1996) and readily explains the occurrence of false-negative bladder washings. Whilst p53 immunohistochemical staining can show heterogeneous distribution within a tumour (Sjogren et al, 1996) we are aware of no data from mutational analyses describing an admixture of wild-type and mutant p53 within the same bladder cancer, and as such heterogeneity is an unlikely cause of false-negative bladder washings.

Two further reports, both published whilst the current study was in progress also establish that p53 mutations can be detected in bladder-washing or urine samples using techniques that do not require prior characterization of the mutation. First, Vet et al (1996) demonstrated p53 abnormalities by SSCP of bladder washings in 6/13 patients with superficial bladder cancer who progressed to invasive disease but in only 1/13 who did not progress. In the second study, Xu et al (1996) identified p53 mutations in 13/28 patients with bladder cancer. Thirty urine samples collected from eight patients whose tumours carried a mutation were collected during 2 years of follow-up. Twenty-four of these demonstrated the same mutation as the primary tumour, the six negative results coinciding with normal appearing cystoscopies. In six cases, despite a negative cystoscopy, mutation was detected in the urine and this finding often heralded the subsequent development of disease recurrence. In this study all mutation detection was performed using direct sequencing, a method possibly less sensitive than SSCP in a mixture of normal and malignant cells (Smith et al, 1992; Wu et al, 1993). That these findings were achieved despite using a technique of limited sensitivity emphasizes that the detection of cancer-associated molecular abnormalities may offer earlier detection of relapsed or recurrent disease than is possible cystoscopically. Whilst the frequency of mutations in the H-*ras* gene in bladder cancer is a contentious issue (Levesque et al, 1993). H-*ras* mutations can be detected in urine sediments of patients with bladder cancer (Levesque et al, 1993; Fitzgerald et al, 1995) and have been shown to allow greater detection of low-grade tumours than urine cytology (Fitzgerald et al, 1995).

A further promising approach to the molecular monitoring of bladder cancer involves the detection of the same microsatellite abnormalities present in bladder tumour samples in corresponding urine samples (Mao et al, 1996). A sensitivity of detecting one malignant cell in a background of $10^2$–$10^3$ normal cells has been reported (Mao et al, 1996) and it has been shown that the persistence or reappearance of a previously detected abnormality following treatment relates to subsequent disease recurrence (Steiner et al, 1997). Collectively these data emphasize that the use of molecular markers in material shed from bladder cancers bears further study.

In conclusion, the current study demonstrates that the detection and characterization of tumour suppressor gene mutations is possible in bladder-washing specimens. It is appropriate to consider the use of such methods for the study of the natural history of bladder cancer as bladder washings are a method of sampling the bladder epitheliums in its entirety. For clinical applicability in disease-monitoring following the treatment of bladder cancer sensitivity will need to be improved. Given the clonal nature of most bladder cancer recurrences (Sidransky et al, 1992) the development of sensitive mutation specific assays for a particular mutation characterized in an individual patient's primary tumour may be an appropriate avenue of further research and prospective evaluation in the follow-up of bladder cancer. Whilst such methods of molecular monitoring of disease are labour- and resource-intensive, they may well become practically achievable given the rapid advances in molecular biological technology and could potentially lead to a reduction in the frequency of invasive procedures.

### ACKNOWLEDGEMENTS

The help of consultant urologists Mr S Prescott, Mr J Fowler and Mr P Bollina in sample collection is gratefully acknowledged. HA Phillips was supported by a Clinical Research Fellowship from the Faculty of Medicine of the University of Edinburgh.

### REFERENCES

Brown R, Clugston C, Burns P, Edlin A, Vasey P, Vojtesek B and Kaye SB (1993) Increased accumulation of p53 protein in cisplatin-resistant ovarian cell lines. *Br J Cancer* **55**: 678–684

Esrig D, Spruck CH, Nichols PW, Chaiwaun B, Steven K, Groshen S, Chen S-C, Skinner DG, Jones PA and Cote R (1993) p53 nuclear protein accumulation correlates with mutations in the p53 gene, tumour grade and stage in bladder cancer. *Am J Pathol* **143**: 1389–1397

Fitzgerald JM, Ramchurren N, Rieger K, Levesque P, Silverman M, Libertino JA and Summerhayes IC (1995) Identification of H-*ras* mutations in urine sediments complements cytology in the detection of bladder tumors. *J Natl Cancer Inst* **87**: 129–133

Fujimoto K, Yamada Y, Okajima E, Kakizoe T, Sasaki H, Sugimura T and Terada M (1992) Frequent association of p53 gene mutation in invasive bladder cancer. *Cancer Res* **52**: 1393–1398

Hermansen DK, Reuter VE, Whitmore WF, Fair WR and Melamed MR (1988) Flow cytometry and cytology as response indicators to M-VAC (methotrexate, vinblastine, doxorubicin and cisplatin). *J Urol* **140**: 1394–1396

Levesque P, Ramchurren N, Saini K, Joyce A, Libertino J and Summerhayes IC (1993) Screening of human bladder tumours and urine sediment for the presence of H-ras mutations. *Int J Cancer* **55**: 785–790

Maier U, Simak R and Neuhold N (1995) The clinical value of urinary cytology: 12 years of experience with 615 patients. *J Clin Pathol* **48**: 314–317

Mao L, Schoenberg M, Scicchitano M, Erozan YS, Merlo A, Schwab D and Sidransky D (1996) Molecular detection of primary bladder cancer by microsatellite analysis. *Science* **271**: 659–662

Nigro JM, Baker SJ, Preisinger AC, Jessup JM, Hostetter R, Cleary K, Bigner SH, Davidson N, Baylin S, Devilee P, Glover T, Collins FS, Weston A, Modali R, Harris CC and Vogelstein B (1989) Mutations in the p53 gene occur in diverse human tumour types. *Nature* **342**: 705–708

Orita M, Suzuki Y, Sekiya T and Hayashi K (1989) Rapid and sensitive detection of point mutations and DNA polymorphisms using the polymerase chain reaction. *Genomics* **5**: 874–879

 © 2000 Cancer Research Campaign

Prosser J, Thompson AM, Granston G and Evans HJ (1990) Evidence that p53 behaves as a tumour suppressor gene in sporadic breast tumours. *Oncogene* **5**: 1573–1579

Phillips HA, Burns D, Prosser J, Howard GCW and Miller WR (1996) The sensitivity of single-stranded conformational polymorphism in mutation detection in a mixed cell population. *Br J Cancer* **73**: 45

Sidransky D, Von Eschenbach A, Tsai YC, Jones P, Summerhayes I, Marshall F, Paul M, Green P, Hamilton SR, Frost P and Vogelstein B (1991) Identification of p53 gene mutations in bladder cancers and urine samples. *Science* **252**: 706–709

Sidransky D, Frost P, Von Eschenbach A, Oyasu R, Preisinger AC and Vogelstein B (1992) Clonal origin of bladder cancer. *N Engl J Med* **326**: 737–740

Sjögren S, Inganas M, Norberg T, Lindgren A, Nordgren H, Holmberg L and Bergh J (1996) The p53 gene in breast cancer: prognostic value of complementary DNA sequencing versus immunohistochemistry. *J Natl Cancer Inst* **88**: 173–182

Smith TA, Whelan J and Parry PJ (1992) Detection of single-base mutations in a mixed population of cells: a comparison of SSCP and direct sequencing. *Genet Anal Tech Applicat* **9**: 143–145

Steiner G, Schoenberg MP, Linn JF, Mao L and Sidransky D (1997) Detection of bladder cancer recurrence by microsatellite analysis of urine. *Nat Med* **3**: 621–624

Vet JA, Witjes JA, Marras SAE, Hessels D, van der Poel HG, Debruyne FMJ and Schalken JA (1996) Predictive value of p53 mutations analyzed in bladder washings for progression of high-risk superficial bladder cancer. *Clin Cancer Res* **2**: 1055–1061

Walther PJ (1992) The role of flow cytometry in the management of bladder cancer. *Hematol Oncol Clin North Am* **6**: 81–98

Winship PR (1989) An improved method for directly sequencing PCR amplified material using dimethyl sulphoxide. *Nucleic Acids Res* **17**: 1266

Wu JK, Ye Z and Darras BT (1993) Sensitivity of single-strand conformation polymorphism (SSCP) analysis in detecting p53 point mutations in tumours with mixed cell populations. *Am J Hum Genet* **52**: 1273–1275

Xu X, Stower MJ, Reid IN, Garner RC and Burns PA (1996) Molecular screening of multifocal transitional cell carcinoma of the bladder using p53 mutations as biomarkers. *Clin Cancer Res* **2**: 1795–1800

*© 2000 Cancer Research Campaign* 
